# Phenotype and genotype of Boerka goats raised in Bali

**DOI:** 10.14202/vetworld.2023.912-917

**Published:** 2023-05-07

**Authors:** I Nyoman Suyasa, I Wayan Suardana, I Gusti Agung Arta Putra, Ni Nyoman Suryani

**Affiliations:** 1 Department of National Research and Innovation Agency, Doctoral Student of The Faculty of Animal Husbandry, Udayana University, Bali, Indonesia; 2 Department of Veterinary Public Health, Faculty of Veterinary Medicine, Udayana University, Bali, Indonesia; 3 Laboratory of Anatomy and Physiology, Faculty of Animal Husbandry, Udayana University, Bali, Indonesia

**Keywords:** Boerka goats, genotype, phenotype, raised in Bali

## Abstract

**Background and Aim:**

Boerka goats are the new breed in Bali targeted at meeting the increasing demand for lamb. They are meat-type goats created by crossing male Boer and female Kacang breeds. This new breed is expected to have good adaptation in a poor environment, such as Kacang goats and produce good quality lamb as Boer goats. Therefore, this study aimed to examine phenotypic and genotypic characteristics of Boerka goats raised in Bali.

**Materials and Methods:**

A total of 16 female Boerka goats at 2 years old collected from a group of livestock farmers in Sanda Village were used as samples. This observational study began with observations of qualitative characteristics and morphometric measurements of goats, followed by polymerase chain reaction (PCR) of the growth hormone (GH) gene using GH5F and GH5R primers. Polymerase chain reaction products were then sequenced and analyzed with the MEGA 11 program.

**Results:**

The results showed that all Boerka goats had stuck-down ears, the heads were predominantly brown in color (62.5%), the body color pattern tends to be white (87.50%), and the tail color pattern was also dominated by white (62.5%). In morphometric terms, the samples were close to Kacang goats based on the body weight, head length, head width, chest width, depth, and circumference, left front leg circumference, ear length, ear width, tail length, tail width, and flank height except for head height, body length, horn length, and shoulder height. Analysis of the GH gene showed that Boerka goats had a nucleotide composition dominated by the purine base guanine (26.5%) and pyrimidine cytosine (31.8%). Furthermore, they formed a separate cluster with a genetic distance of 14.1% to the Anhui White breed from China, the Tibetan breed, and the Swiss Saanen breed.

**Conclusion:**

Boerka goats raised in Bali have phenotypes, including qualitative characteristics and morphometric measurements close to Kacang goats. As meat-type goats, they also form a separate cluster distinct from similar types worldwide.

## Introduction

The tropical climate condition in Indonesia is an important factor in supporting the development of goats’ farming [1]. Goats are generally raised from a scale of 2–5 heads/farmer and can be upgraded to 5–10 heads/farmer. Several advantages and economic potential of goats’ farming include the body being relatively small, early sexual maturity, easy to maintain, minimal land requirement, relatively small business capital investment, and being easily marketed [2]. Some goats, including Kacang, Bligon, Etawah grade, Gembrong, Marica, and Samosir can be found in Indonesia as germplasm [3]. On August 23, 2019, the Bali government imported about 100 Boerka goats consisting of 12 males and 82 females from the Sei Putih Goat Research Workshop (Lolit Goat), North Sumatra. The goats were in the form of direct assistance from the Research and Development Center for Animal Husbandry, Bogor. More specifically, Boerka goats as an innovation, can be created by cross-breeding male Boer and female Kacang breeds. They were introduced to meet the lamb demand in this area due to the imbalance of the supply by Kacang and Etawah grades. This policy was stated in the decree of the Ministry of Agriculture No. 472/Kpts/RC.040/06/2018 [4], which stipulates the Tabanan Regency, Bali Province, as a center for the goats’ development area.

Boerka goats are expected to have two advantages as a combination of the two parents, namely, the superior lamb quality produced from male Boer and the wide adaptability in the tropical-wet environment as well as the ability to live on a minimalist diet inherited from female Kacang [5]. The development of Boerka goats in Bali was centered in Sanda Village, located in Papuan District, Tabanan Regency. According to data collected between 2019 and 2022 from the leader of the farmer group in Sanda, the population of Boerka goats is 217 heads consisting of 12 males, 88 females, and 131 goatlings. However, no information has been published about their phenotype and genotype.

This study aims to examine phenotypic appearance of Boerka goats in the form of qualitative characteristics and morphometrics data, as well as their genetic analysis related to the growth hormone (GH).

## Materials and Methods

### Ethical approval

The study protocol was approved by the Ethics Committees on Animal Experiments, Faculty of Veterinary Medicine-Udayana University, Certificate No. No. B/44/UN14.2.9/PT.01.04/2023.

### Study period and location

The study was conducted from June to September 2022 at the Integrated Laboratory of the Study Program of Biotechnology, Gadjah Mada University, and the Laboratory of Veterinary Public Health, Faculty of Veterinary Medicine, Udayana University.

### Qualitative characteristics and morphometrics

A total of 16 female Boerka goats collected from a group of livestock farmers in Sanda Village aged 2 years old were used as samples. This selection was based on the consideration that female goats population was more predominant in the study area, and the number of male goats is limited. Observations were made on the qualitative appearance, including the ear shape, as well as the head, body, and tail color pattern. Morphometric measurements were also carried out on several parameters, including head length, width, and height, chest width, chest depth, shoulder height, chest circumference, body length, and cannon circumference of the left front leg [6]. Data measurement results were analyzed descriptively and presented in the form of mean, standard deviation, and coefficient of variation [7].

### Growth hormone gene analysis

#### Blood collection of the samples

A total of 2 mL blood from female Boerka goats was taken from the jugular vein using a 5 mL venoject-tube which had been filled with ethylenediaminetetraacetic acid anticoagulant. The samples were then brought to the laboratory in an ice box for the next test.

#### Extraction of DNA and polymerase chain reaction (PCR)

DNA was extracted from blood samples using QIAamp DNA Mini Kits (Qiagen, Germany) according to the manufacturer’s instructions as described previously [8–11]. The GH gene was amplified using MyTaq HS Red Mix (Bioline Reagents, UK) on Thermocycler Eppendorf Mastercycler personal/PTC 100, USA. The PCR program was carried out in 25 μL reaction volumes containing 1 μL DNA template (300 ng/μL), 12.5 μL PCR MyTaq HS Red Mix 2×, and 2 μL (10 pmol/μL) of each primer. The primers used in this study were GH5F 5-GCTGCTCCTGAGGGCCCTTC-‘3 and GH5R 5-CATGACCCTCAGGTACGTCTCCG-‘3 [12]. The PCR amplification was conducted with initial DNA denaturation at 94°C for 5 min, followed by 30 cycles of denaturation at 94°C for 1 min, annealing at 58°C for 30 s, elongation at 72°C for 5 min, and final extension at 72°C for 5 min. Furthermore, 5 μL PCR product was analyzed by electrophoresis (Bio-Rad, USA) in 1% agarose (Gibco BRL, USA) gel, at 100 volts for 30 min, followed by staining with FluoroVue Nucleic Acid solution (5 μL/50 mL, Smobio, Germany). The gel was visualized by ultraviolet trans-illumination and recorded by a digital camera FE-270 7.1 megapixels.

#### Sequencing and phylogenetic analysis

The sequencing of the GH gene was conducted using a genetic analyzer (ABI Prism 3130 and 3130 xlGeneticAnalyzer, USA) at 1^st^ BASE Pte Ltd, Singapore, through PT. Genetica Science Service, Jakarta. The sequencing also used both primers, namely, GH5F and GH5R, while the sequences were edited to exclude the PCR primer binding sites and manually corrected using MEGA 11 version software (https://www.megasoftware.net/). The GH gene sequences of Boerka goats were compared automatically using the BLAST against several sequences of goats available in databanks (http://www.ncbi.nlm.nih.gov/) [13]. Furthermore, the phylogenetic analysis was constructed using the unweighted pair group method and arithmetic mean algorithm [14, 15].

## Results

### Qualitative characteristics and morphometrics

The results showed that Boerka goats raised in Bali were intensively in a cage and the feed given was usually foraged around their cage. The phenotypic data, including the qualitative characteristics and quantitative morphometrics are shown in Tables-1 and 2.

**Table-1:** Quantitative characteristics of female Boerka goat raised in Bali.

Quantitative traits	n (%)
Ear shape pattern	
Stand up	0 (0/16)
Dangling down	100 (16/16)
Head color pattern	
White brown	12.5 (2/16)
Brown	62.5 (10/16)
Black	25 (4/–16)
Body color pattern	
White	87.50 (14/16)
White brown	12.5 (2/16)
Tail color pattern	
Light brown	12.5 (2/16)
Brown	25 (4/–16)
White	62.5 (10/16)

**Table-2:** Morphometrics of female Boerka goat raised in Bali.

Qualitative traits	Measurement (mean [SD])
Body weight (kg)	29.04 (±4.51)
Head length (cm)	17.50 (±1.89)
Head width (cm)	10.53 (±0.35)
Head height (cm)	11.08 (±1.55)
Chest width (cm)	15.80 (±2.24)
Deep of chest (cm)	27.93 (±1.50)
Chest circle (cm)	68.85 (±5.15)
Body length (cm)	63.07 (±3.48)
Horn length (cm)	12.46 (±3.71)
Front left-right canon circle (cm)	8.054 (±1.25)
Ear length (cm)	18.62 (±1.90)
Ear width (cm)	7.73 (±0.53)
Tail length (cm)	14.54 (±3.91)
Tail width (cm)	3.81 (±0.48)
Shoulder height (cm)	58.79 (±3.28)
Hip height (cm)	60.39 (±4.09)

SD=Standard deviation

Table-1 shows that all Boerka goats have downward protruding ears, their head color pattern is dominated by brown (62.5%), the body color pattern tends to be white (87.50%), and the tail color pattern is also dominated by white (62.5%).

The morphometrics data in Table-2 showed a slight difference from the results of Nuraini and Asmarhansyah [6], showing that adult male and female Boerka goats had a body length of 60 (± 2.45 cm) and 71.5 (± 2.12 cm); shoulder height 55.11 (± 3.86 cm) and 63.5 (± 3.54 cm); hip height 59 (± 2.74 cm) and 68.5 (± 2.12 cm); chest depth 32.11 (± 2.32 cm) and 40 (± 2.83 cm); and chest width 11.56 (± 0.88 cm) and 16.5 (± 0.71 cm), respectively. This shows that the morphometric measurements of Boerka goats kept in Bali are smaller in size than those kept at KP Petaling, Bangka Belitung Regency.

#### Boerka goats GH gene analysis

**Figure-1:**
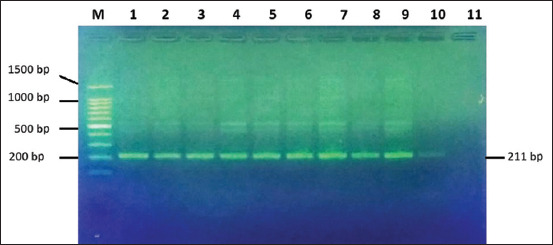
A polymerase chain reaction of the growth hormone gene in the Boerka goat sample with the primers GH5F and GH5R on 1.5% agarose. M: Maker 100 bp DNA ladder (Promega), 1–10: Boerka sample no 1–10. 11: Negative control.

The amplification of the GH gene with the primers GH5F and GH5R was successfully carried out as indicated by the appearance of a single PCR product at position 211 bp. The amplification result is shown in Figure-1.

Figure-1 shows the amplification of the GH gene using primers GH5F and GH5R with a size of 211 bp. This primer spans from intron 4 (49 bp) to exon 5 (162 bp) [12].

## Discussion

The qualitative characteristics of Boerka goats raised in Bali are in line with the results of a previous study conducted in 2016 at the Experimental Station of White Sei Goat Research in Deli Serdang, Sumatra. The results showed that Boerka goats generally have a combination of white, brown, and black colors [16], with the white color having the largest surface area on the body parts. Many brown colors are found on the neck and head, while the white color is dominant on the body, legs, and tail. The color is a combination of Kacang goats’ color which spreads widely, followed by the pattern of Boer goats [16]. Furthermore, phenotypic markers have been widely used both in basic genetics and in practical breeding programs because they are easy to observe and differentiate. Measurements of body parameters are also commonly used to predict the origin of livestock breeds. Body measurements are very useful for determining the origin and phylogenetic relationships between different species, breeds, and types of livestock. Several studies used criteria such as body measurements to differentiate groups of Marica goats outside their habitat [17] and the Etawah crossbreed (PE) in Muaro Jambi [18].

The difference in morphometry between Boerka goats raised within and outside of Bali, specifically those kept at the Petaling Bangka Belitung KP, is presumably caused by variations in the rearing system and environmental conditions. According to Suryo [19], the environment is the biggest factor that affects phenotypic appearance. Animals with the same genotype can show distinct phenotypes when the environment for the two genotypes is different.

Besides environmental factors, genetics also affect phenotypic performance, specifically the GH gene which plays an important role in various biological processes of growth, metabolism, lactation, and reproduction of animals [20, 21]. Growth hormone is a peptide hormone, and its production is controlled by genes. The GH gene is mapped on the short arm of chromosome 19 (*Capra hircus* 19q22) [22], which consists of five exons and four introns (Accession: D00476) [23]. This hormone is released from the anterior lobe of the pituitary gland, while the major functions include stimulating muscle and bone growth, through its action with insulin-like growth factor [24], as well as milk production [25]. However, molecular changes in the gene coding due to mutations can cause changes in the structure of the protein produced, affecting the effectiveness of the hormone [26].

The complete analysis of the GH gene nucleotide to control the expression of the GH is very important as variation/polymorphism of this gene will greatly affect the growth traits of animals, in this case, goats. To analyze the GH gene, the nucleotide sequence of Boerka goats was aligned against other genes available in the Genbank and the results are presented in Table-3.

**Table-3:** Alignment of the nucleotide sequence of the GH gene in Boerka goats from Bali with some of the available GH gene nucleotides in the gene bank.

#Boerka_GH-1	CTG	CTC	CTG	AGG	GCC	CTT	CGG	CCT	CTC	TGT	---	CTC	TCC	[39]
#Boerka_GH-2	…	…	…	…	…	…	…	…	…	…	…	…	…	[39]
#Boerka_GH-3	…	…	…	…	…	…	…	…	…	…	…	…	…	[39]
#Boerka_GH-4	…	…	…	…	…	…	…	…	…	…	…	…	…	[39]
#Boerka_GH-5	…	…	…	…	…	…	…	…	…	…	…	…	…	[39]
#*Capra_hircus*_(JX570671)	G..	TCT	A..	..A	AG.	TGA	A..	A.C	TGG	A.G	AAG	GCA	…	[39]
#*Capra_hircus*_(JF896274)	G..	TCT	A..	..A	AG.	TGA	A..	A.C	TGG	A.G	AAG	GCA	…	[39]
#*Capra_hircus*_(JF813118)	G..	TCT	A..	..A	AG.	TGA	A..	A.C	TGG	A.G	AAG	GCA	…	[39]
#Goat_mRNA_gh_(Y00767)	G..	TCT	A..	..A	AG.	TGA	A..	A.C	TGG	A.G	AAG	GCA	…	[39]
#Boerka_GH-1	CTC	CCT	TG-	-GC	AGG	AGC	TGG	AAG	ATG	TTA	CCC	CCC	GGG	[78]
#Boerka_GH-2	…	…	…	…	…	…	…	…	…	…	…	…	…	[78]
#Boerka_GH-3	…	…	…	…	…	…	…	…	…	…	…	…	…	[78]
#Boerka_GH-4	…	…	…	…	…	…	…	…	…	…	…	…	…	[78]
#Boerka_GH-5	…	…	…	…	…	…	…	…	…	…	…	…	…	[78]
#*Capra_hircus*_(JX570671)	TGG	C	A	T	G	…	…	…	…	…	…	…	…	[78]
#*Capra_hircus*_(JF896274)	TGG	C	A	T	G	…	…	…	…	…	…	…	…	[78]
#*Capra_hircus*_(JF813118)	TGG	C	A	T	G	…	…	…	…	…	…	…	…	[78]
#Goat_mRNA_gh_(Y00767)	TGG	C	A	T	G	…	…	…	…	…	…	…	…	[78]
#Boerka_GH-1	CTG	GGC	AGA	TCC	TCA	AGC	AGA	CCT	ATG	ACA	AAT	TTG	ACA	[117]
#Boerka_GH-2	…	…	…	…	…	…	…	…	…	…	…	…	…	[117]
#Boerka_GH-3	…	…	…	…	…	…	…	…	…	…	…	…	…	[117]
#Boerka_GH-4	…	…	…	…	…	…	…	…	…	…	…	…	…	[117]
#Boerka_GH-5	…	…	…	…	…	…	…	…	…	…	…	…	…	[117]
#*Capra_hircus*_(JX570671)	…	…	…	…	…	…	…	…	…	…	…	…	…	[117]
#*Capra_hircus*_(JF896274)	…	…	…	…	…	…	…	…	…	…	…	…	…	[117]
#*Capra_hircus*_(JF813118)	…	…	…	…	…	…	…	…	…	…	…	…	…	[117]
#Goat_mRNA_gh_(Y00767)	…	…	…	…	…	…	…	…	…	…	…	…	…	[117]
#Boerka_GH-1	CAA	ACA	TGC	GCA	GTG	ACG	ACG	CGC	TGC	TCA	AGA	ACT	ACG	[156]
#Boerka_GH-2	…	…	…	…	…	…	…	…	…	…	…	…	…	[156]
#Boerka_GH-3	…	…	…	…	…	…	…	…	…	…	…	…	…	[156]
#Boerka_GH-4	…	…	…	…	…	…	…	…	…	…	…	…	…	[156]
#Boerka_GH-5	…	…	…	…	…	…	…	…	…	…	…	…	…	[156]
#*Capra_hircus*_(JX570671)	…	…	…	…	…	…	…	…	…	…	…	…	…	[156]
#*Capra_hircus*_(JF896274)	…	…	…	…	…	…	…	…	…	…	…	…	…	[156]
#*Capra_hircus*_(JF813118)	…	…	…	…	…	…	…	…	…	…	…	…	…	[156]
#Goat_mRNA_gh_(Y00767)	…	…	…	…	…	…	…	…	…	…	…	…	…	[156]
#Boerka_GH-1	GTC	TGC	TCT	CCT	GCT	TCC	GGA	AGG	ACC	TGC	ACA	AGA	CGG	[195]
#Boerka_GH-2	…	…	…	…	…	…	…	…	…	…	…	…	…	[195]
#Boerka_GH-3	…	…	…	…	…	…	…	…	…	…	…	…	…	[195]
#Boerka_GH-4	…	…	…	…	…	…	…	…	…	…	…	…	…	[195]
#Boerka_GH-5	…	…	…	…	…	…	…	…	…	…	…	…	…	[195]
#*Capra_hircus*_(JX570671)	…	…	…	…	…	…	…	…	…	…	…	…	…	[195]
#*Capra_hircus*_(JF896274)	…	…	…	…	…	…	…	…	…	…	…	…	…	[195]
#*Capra_hircus*_(JF813118)	…	…	…	…	…	…	…	…	…	…	…	…	…	[195]
#Goat_mRNA_gh_(Y00767)	…	…	…	…	…	…	…	…	…	…	…	…	…	[195]
#Boerka_GH-1	AGA	CGT	ACC	TGA	GGG	TCA	TGA	[216]						
#Boerka_GH-2	…	…	…	…	…	…	…	[216]						
#Boerka_GH-3	…	…	…	…	…	…	…	[216]						
#Boerka_GH-4	…	…	…	…	…	…	…	[216]						
#Boerka_GH-5	…	…	…	…	…	…	…	[216]						
#*Capra_hircus*_(JX570671)	…	…	…	…	…	…	…	[216]						
#*Capra_hircus*_(JF896274)	…	…	…	…	…	…	…	[216]						
#*Capra_hircus*_(JF813118)	…	…	…	…	…	…	…	[216]						
#Goat_mRNA_gh_(Y00767)	…	…	…	…	…	…	…	[216]						

GH=Growth hormone

Table-3 shows the variation in the nucleotide sequence between the GH gene of Boerka goats and others, including *C. hircus* (JX570671) as a meat-type goat from the Anhui white breed in China, *C. hircus* (JF896274) and *C. hircus* (JF813118) from Tibetan, as well as Goat mRNA GH (Y00767) as a “Saanen” type goat originating from the Saanen valley, also known as a milk producer. The analysis results showed that Boerka goats have different GH gene nucleotide compositions compared to other goat breeds raised outside Bali, but no nucleotide variations were found. These results indicated that in terms of the GH gene appearance, Boerka goats in Bali are classified as monomorphic because no mutations have been found in the form of deletions, transitions, or transversions. The nucleotide composition of Boerka goats’ GH gene compared to other breeds is presented in Table-4.

**Table-4:** The nucleotide composition of the Boerka goat’s GH gene compared to other goat breeds.

Breeds	T(U)	C	A	G	Total
Boerka GH-1	20.4	31.8	21.3	26.5	211
Boerka GH-2	20.4	31.8	21.3	26.5	211
Boerka GH-3	20.4	31.8	21.3	26.5	211
Boerka GH-4	20.4	31.8	21.3	26.5	211
Boerka GH-5	20.4	31.8	21.3	26.5	211
*Capra hircus* (JX570671)	18.1	26.4	25.5	30.1	216
*Capra hircus* (JF896274)	18.1	26.4	25.5	30.1	216
*Capra hircus* (JF813118)	18.1	26.4	25.5	30.1	216
Goat mRNA GH (Y00767)	18.1	26.4	25.5	30.1	216

GH=Growth hormone

Based on the data in Table-4, the GH gene in Boerka goats was more dominated by purine bases in the form of guanine (G) at 26.5% and pyrimidine bases, namely, cytosine (C) at 31.8%. In contrast, other goats were dominated by purine G at 30.1% and pyrimidine C at 26.4%. The differences in the nucleotide composition between Boerka and other goats can be used as basis for determining the genetic distance, as shown in Table-5.

**Table-5:** Genetic distance based on the GH gene between Boerka goats and several other goat breeds.

GH gene of goat strains	Boerka GH-1	Boerka GH-2	Boerka GH-3	Boerka GH-4	Boerka GH-5	*Capra hircus* (JX570671)	*Capra hircus* (JF896274)	*Capra hircus* (JF813118)	Goat mRNA GH (Y00767)
Boerka GH-1									
Boerka GH-2	0.000								
Boerka GH-3	0.000	0.000							
Boerka GH-4	0.000	0.000	0.000						
Boerka GH-5	0.000	0.000	0.000	0.000					
*Capra hircus* (JX570671)	0.141	0.141	0.141	0.141	0.141				
*Capra hircus* (JF896274)	0.141	0.141	0.141	0.141	0.141	0.000			
*Capra hircus* (JF813118)	0.141	0.141	0.141	0.141	0.141	0.000	0.000		
Goat mRNA GH (Y00767)	0.141	0.141	0.141	0.141	0.141	0.000	0.000	0.000	

GH=Growth hormone

**Figure-2:**
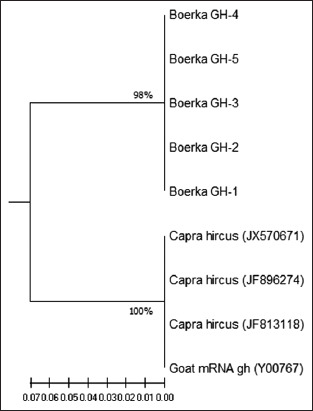
Phylogenetic tree of the boerka goat against other goat breeds. The phylogram was constructed based on the unweighted pair group method and arithmetic mean algorithm (Saitou and Nei, 1987) from growth hormone gene nucleotides (211 nt in size). The numbers on the phylogram branch indicate the bootstrap value (%) with 500 replications.

The data in Table-5 shows that there is a genetic distance between Boerka and other goats. In addition, there is a genetic distance among Boerka goats. The analysis results showed that there was an identical GH gene among Boerka goats raised in Bali, but they show genetic distance to others raised externally, such as *C. hircus* (JX570671), *C. hircus* (JF896274), *C. hircus* (JF813118), and Goat mRNA GH (Y00757). The genetic distance was calculated to be 14.1% which translates to a difference of 141/1000 nucleotides. This difference in genetic distance culminated in the grouping of Boerka goats into a different cluster, as shown in Figure-2.

Figure-2 shows that Boerka goats raised in Bali form a separate cluster from others with a bootstrap value of 98%. Meanwhile, *C. hircus* (JX570671), a meat-type goat from the Anhui white breed in China, *C. hircus* (JF896274), and *C. hircus* (JF813118) from Tibetan, as well as Goat mRNA GH (Y00767) as a “Saanen” type goat originating from the Saanen valley, western Switzerland, form a separate cluster with a 100% bootstrap value. These results indicated that Boerka goats indeed have different clusters and the genetic difference is closely related to phenotypic performances.

## Conclusion

The phenotype of Boerka goats, including the qualitative characteristics and morphometric measurements, is close to Kacang goats as their parent. Furthermore, the GH gene, as one of the principal genetic markers, showed a nucleotide composition dominated by the purine base G (26.5%) and pyrimidine C (31.8%). Based on the results, Boerka goats formed a separate cluster with a genetic distance of 14.1% from other meat-type goats raised outside Bali.

## Authors’ Contributions

INS: Conceptualized and designed the study and collected data. IWS: Conceptualization, methodology, data curation, resources, project administration, and writing - review and editing. IGAAP and NNS: Designed and formulated material in the laboratory and supervised data. INS and IWS: Drafted, edited, and critically revised the manuscript. All authors have read, reviewed, and approved the final manuscript.
